# Analysis of *TRIOBP* gene in non-syndromic deafness: A case report

**DOI:** 10.1097/MD.0000000000040435

**Published:** 2024-11-08

**Authors:** Hong Zhou, Gang Guo, Jianjun Gao, Hong Duan

**Affiliations:** a Affiliated Hospital of Inner Mongolia Medical University, Hohhot, P.R. China; b Department of Otolaryngology and Head and Neck Surgery, Affiliated Hospital of Inner Mongolia Medical University, Hohhot, P.R. China.

**Keywords:** deafness gene, hereditary deafness, pathogenic variation, *TRIOBP* gene

## Abstract

**Rationale:**

Through family investigation, the genetic map was drawn and audiological characteristics were analyzed. High-throughput sequencing was used to screen the deafness genes of the proband. Sanger sequencing was used to verify the suspected pathogenic sites in the family.

**Patient concerns:**

Identify the causes of hearing loss and treatment options.

**Diagnoses:**

Bilateral moderate to severe sensorineural deafness.

**Interventions:**

After completing the examination, the patient was recommended to wear a hearing aid or do a cochlear implant, but the patient was not treated for personal reasons.

**Outcomes:**

All 8 patients in this family were nonsyndromic deafness. The proband had a compound heterozygous mutation of c.A4484T/c.A4510G in the *TRIOBP* gene, and the patient II-6 had a heterozygous mutation of c.A4484T in the *TRIOBP* gene. A complex heterozygous mutation of *TRIOBP* gene c.A4510G/c.G59T was found in II-7, but no reports of pathogenicity of these mutations were found in relevant literatures and databases. In addition, patients II-6, III-4, and III-6 had heterozygous mutations of *CHD7* gene c.T2615C and C.3202-5T >C, and patients II-6 and III-4 also had heterozygous mutations of *CHD23* gene c.G5312A and c.C6250T.

**Lessons:**

In this study, a new locus of the *TRIOBP* gene was found, which enriched the gene mutant spectrum and clarified the pathogenic gene of the proband. However, the etiology of deafness in other members of the family needs to be further analyzed.

## 1. Introduction

Hearing loss is a prevalent sensory disorder, with an incidence rate of approximately 1‰ to 3‰ in newborns. International data indicate that approximately 60% of hearing loss cases are associated with genetic factors.^[1]^ Recent statistics in China show that genetic hearing loss accounts for 67% of severe or profound cases.^[2]^ Genetic hearing loss is a highly heterogeneous disease and can be classified into syndromic hearing loss (30%) and non-syndromic hearing loss (70%) based on the presence or absence of other systemic conditions. Genetic hearing loss primarily involves 4 modes of inheritance: autosomal dominant (Autochromatic dominant hereditary non-syndromic deafness, DFNA, 15%–20%), autosomal recessive (Autochromatic recessive hereditary non-syndromic deafness, DFNB, 80%), X-linked (X-linked non-syndromic hereditary deafness, DFN X-linked; Y-linked non-syndromic hereditary deafness, DFN Y-linked, 1%), and mitochondrial (1%). Non-syndromic hearing loss, which accounts for the majority, with 75%–80% attributed to autosomal recessive inheritance, often manifests as severe congenital sensorineural hearing loss.^[3]^

For individuals with genetic hearing loss, diagnosis can be established through clinical manifestations, family history, audiological examinations, and genetic testing results. Currently, molecular etiology can be determined in approximately 40% of the Chinese population with hearing loss through genetic diagnosis. Considering that genetic hearing loss accounts for 60% of all cases, an estimated 66% of genetic hearing loss cases have received a definitive diagnosis. However, the molecular etiology of 34% of genetic hearing loss patients remains to be elucidated through the development of higher throughput hearing loss gene testing chips or the identification of novel hearing loss genes.

Chinese researchers have developed various techniques, including restriction enzyme analysis, real-time quantitative polymerase chain reaction, high-performance liquid chromatography, microarrays, Sanger sequencing, and next-generation sequencing, to establish a series of genetic testing methods for hearing loss. These methods encompass rapid screening for hotspot mutations, high-density hearing loss gene mutation detection chips, comprehensive sequencing of key hearing loss genes, and large-scale sequencing covering all known hearing loss genes, thereby facilitating the clinical application of hearing loss gene diagnosis and screening.^[4]^

The *TRIOBP* gene represents an important genetic factor causing non-syndromic hearing loss. It is also known as TRIO and F-actin-binding protein, alternatively referred to as *TARA*, *TAP68*, and *HRIHFB2122*. Located on chromosome 22q13.1, the *TRIOBP* gene spans a total length of 79,509 base pairs and comprises 26 exons. *TRIOBP* gene expression is widely observed in eukaryotic cells and plays a critical role in cellular cytoskeletal reorganization, proliferation, and migration processes.^[5]^

In 2006, Shahin et al^[6]^ and Riazuddin et al^[7]^ first identified the association between the *TRIOBP* gene and non-syndromic hearing loss. To further investigate the genetic mechanism of this gene, our study selected a non-syndromic hearing loss pedigree for genetic research. Whole-genome sequencing was conducted, and the pathogenicity of *TRIOBP* gene compound heterozygous mutations in the pedigree was confirmed through Sanger sequencing.

The aim of this study is to conduct a detailed analysis of the *TRIOBP* gene to uncover its specific role in non-syndromic hearing loss. By thoroughly studying the types, locations, and functional impact of *TRIOBP* gene mutations, we can gain a better understanding of its significance in the pathogenesis of hearing loss.

Furthermore, additional techniques and methods, such as functional experiments, construction of cellular models, and expression analysis, will be employed to further validate the functional role of the *TRIOBP* gene and its association with non-syndromic hearing loss. Through these experiments, we aim to gain deeper insights into the specific regulatory mechanisms of the *TRIOBP* gene in the pathogenesis of hearing loss, providing a more scientific and accurate basis for diagnosis and treatment.

In conclusion, this study will conduct an in-depth investigation into the genetic mechanisms and functional roles of the *TRIOBP* gene in non-syndromic hearing loss. It will provide new evidence for understanding the molecular mechanisms underlying hearing loss and offer important scientific foundations for relevant clinical diagnosis and treatment.

## 2. Methods

### 2.1. Patients and samples

**Figure 1.**
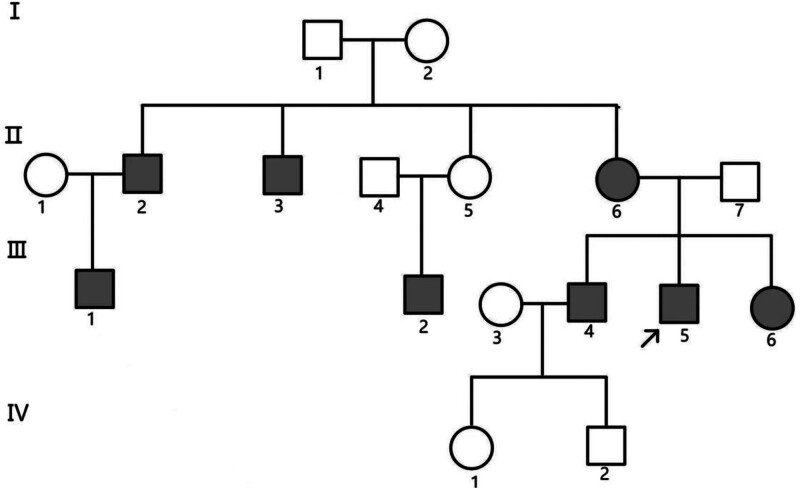
Pedigree diagram: Proband (III-5) is indicated by an upward arrow (↗); Blacking indicates a hearing loss.

This study was approved by the Ethics Committee of YKD202202010. Five milliliters of peripheral blood were obtained from the proband and their family and written informed consent was obtained from the patient’s parents or legal guardians. Proband and family members undergo systemic examination and exclude patients with syndromic deafness. Genetic family maps are drawn based on the results of consultation and audiology (Fig. 1). All patients undergo specialist examination, including pure tone audiometry and acoustic impedance. All patients and their families received genetic counseling, and all data from this study were analyzed anonymously.

### 2.2. Exome sequencing

Genomic Deoxyribonucleic acid from family members (father and mother) and patient was extracted from peripheral blood leucocytes using the DNeasy kit (Qiagen), according to the manufacturer’s instructions. Whole exomes were captured (MyGenostics Inc., Beijing, China) and sequenced on Illumina NovaSeq 6000 series sequencer (PE150). Quality control filters were applied to remove reads with low quality. Bioinformatics analysis was performed using an in-house pipeline that included genome alignment (human reference genome hg19, National Center for Biotechnology Information) with the Burrows-Wheeler Aligner (BWAMEM). We used the online system independently developed by Chigene (www.chigene.org) to annotate database based MAFs and ACMG practice guideline-based pathogenicity of every yielded gene variant. The variants with a minor allele frequency of < 0.05 in population databases, such as 1000 genome, ESP6500, dbSNP, EXAC, and in-house database (MyGenostics), expected to affect protein coding/splicing or present in the Human Gene Mutation Database, were included in the analysis. For CNV calling, SAM tools was used to calculate the every-coding-region total bases. The GATK command was used to obtain the average mean depth of CCDS (Consensus Coding Sequence) regions. R was then used to calculate the ratio of every sample compared with other samples’ mean ratio, and ggplot was used to visualize results. A ratio >1.4 was assigned as a duplication, and <0.6 was assigned as a deletion. To reduce false positives, only deletions or duplications of 2 consecutive exons were identified as true variants.

### 2.3. Bioinformatics analysis

To explain the pathological mechanisms of Non-Syndromic deafness. Our study predicted the Protein interaction network by online software of GeneMANIA. Genes were overlaid onto pathways defined by the Kyoto Encyclopedia of Genes and Genomes (KEGG), Uniprot, and Gene Ontology Consortium (GO) using the R package cluster Profiler. A pathway was considered altered in a given sample if at least 1 gene in the pathway contained a mutation.

### 2.4. Conservative and in silico analysis

Domains of *TRIOBP* were identified based on the National Center for Biotechnology Information Conserved Domain Database (https://www.ncbi.nlm.nih.gov/Structure/cdd/wrpsb.cgi). Multiple sequence alignment of *TRIOBP* was performed using the ClustalW program. Three-dimensional structural models of *TRIOBP* were predicted by the Swiss-model web tool (http://swissmodel.expasy.org/ interactive). Protein structure images were generated using the PDB file and PyMOL (https://pymol.org/2/). Demonstrate hydrogen bonds in proteins using Pymol to predict changes in mutant stability.

## 3. Results

### 3.1. Patient and clinical information

Through investigation, there were 17 individuals in 4 generations with autosomal recessive inheritance. The proband was a male 31 year old with postlingual deafness. Family history investigation revealed that individuals II-2, II-3, II-6, III-1, III-2, III-4, III-5, and III-6 in this family all have non-syndromic hearing loss, with III-5 being the proband. Patient II-3 experienced complete deafness in the left ear after an external injury at the age of 30 (Fig. 1).

### 3.2. Audiological examination

The proband’s tympanograms for both ears showed type As. The tympanometric peak values were 0.26 for the left ear and 0.20 for the right ear. Acoustic reflex thresholds were not elicited at 0.5 kHz, 1 kHz, 2 kHz, and 4 kHz. Pure-tone audiometry results showed that the average air conduction thresholds for the left ear were 65 dBHL at 0.5 kHz, 1 kHz, 2 kHz, 4 kHz, and 8 kHz, while for the right ear, they were 60 dBHL at the same frequencies (Table 1).

**Table 1:** Behavioral audiometry results (dB HL).

Frequency (Hz)	250	500	1000	2000	4000
II-6’s right ear hearing threshold	90	100	95	100	100
II-6’s left ear hearing threshold	90	100	100	100	100
III-4’s right ear hearing threshold	70	70	75	80	85
III-4’s left ear hearing threshold	60	65	70	75	60
III-5’s right ear hearing threshold	55	50	55	65	65
III-5’s left ear hearing threshold	65	65	65	65	65
III-6’s right ear hearing threshold	55	60	85	95	90
III-6’s left ear hearing threshold	65	80	100	100	95

### 3.3. Genetic sequencing and protein structure prediction results

**Figure 2.**
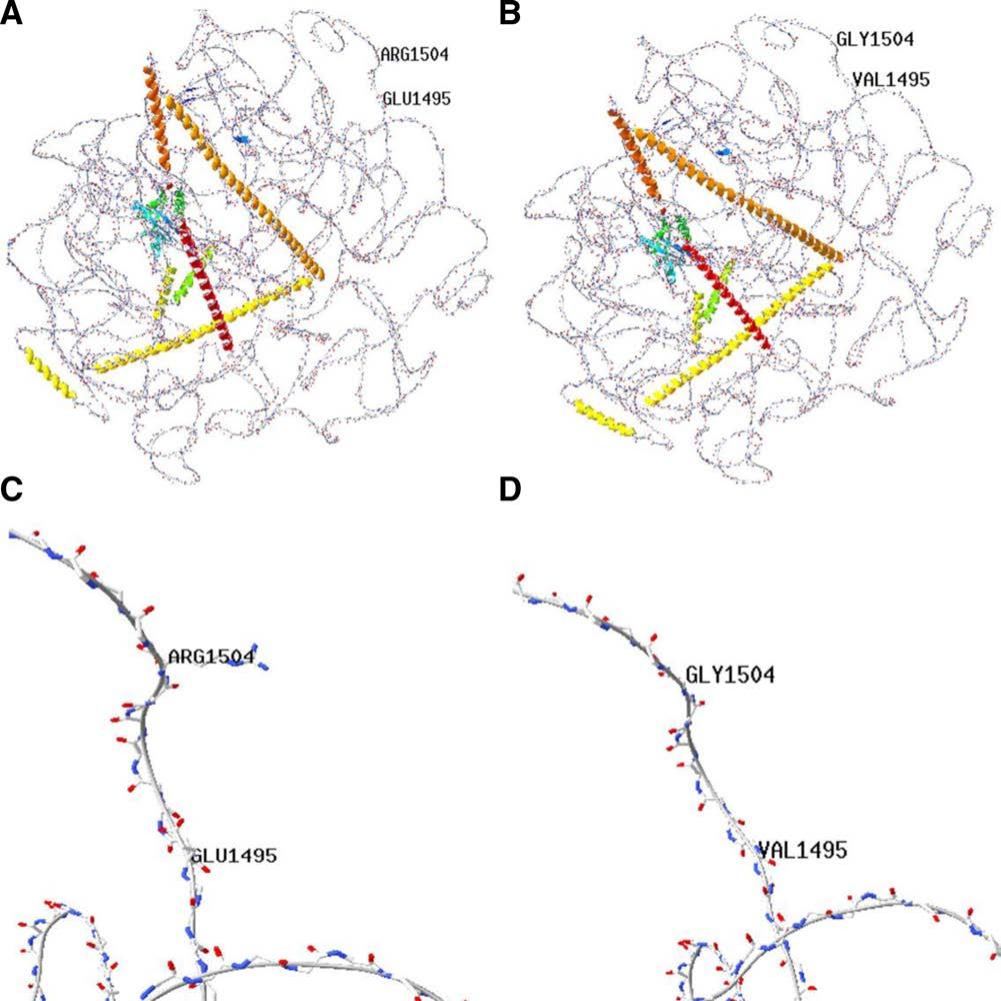
Difference between the wide-type and mutant protein (c.A4484T and c.A4510G) of TRIOBP (regional model). (A) Overall diagram of wild-type protein; (B) overall diagram of mutant protein; (C and D) details of wild protein. At position 1495, the polar, hydrophilic amino acid GLU is mutated to the nonpolar, hydrophobic amino acid VAL (as shown in D). At position 1504, the polar, positively charged amino acid ARG is mutated to the polar, uncharged amino acid GLY (as shown in D).

Through whole-exome sequencing, compound heterozygous mutations in the *TRIOBP* gene (NM_001039141) were detected in the proband (III-5): c.A4484T/p.E1495V (dbSNP: rs183455182) and c.A4510G/p.R1504G (dbSNP: rs374991119), both being missense mutations. Protein structure homology modeling analysis was performed using the online Swiss-Model software. The mutation at amino acid position 1495 changed from a negatively charged GLU (polar hydrophilic) (Fig. 2A and C) to a nonpolar hydrophobic amino acid, VAL (Fig. 2B and D). The mutation at amino acid position 1504 changed from a positively charged ARG (polar) (Fig. 2A and C) to a neutral GLY (Fig. 2B and D). These changes in amino acid properties may affect the protein structure and consequently its function.

**Figure 3.**
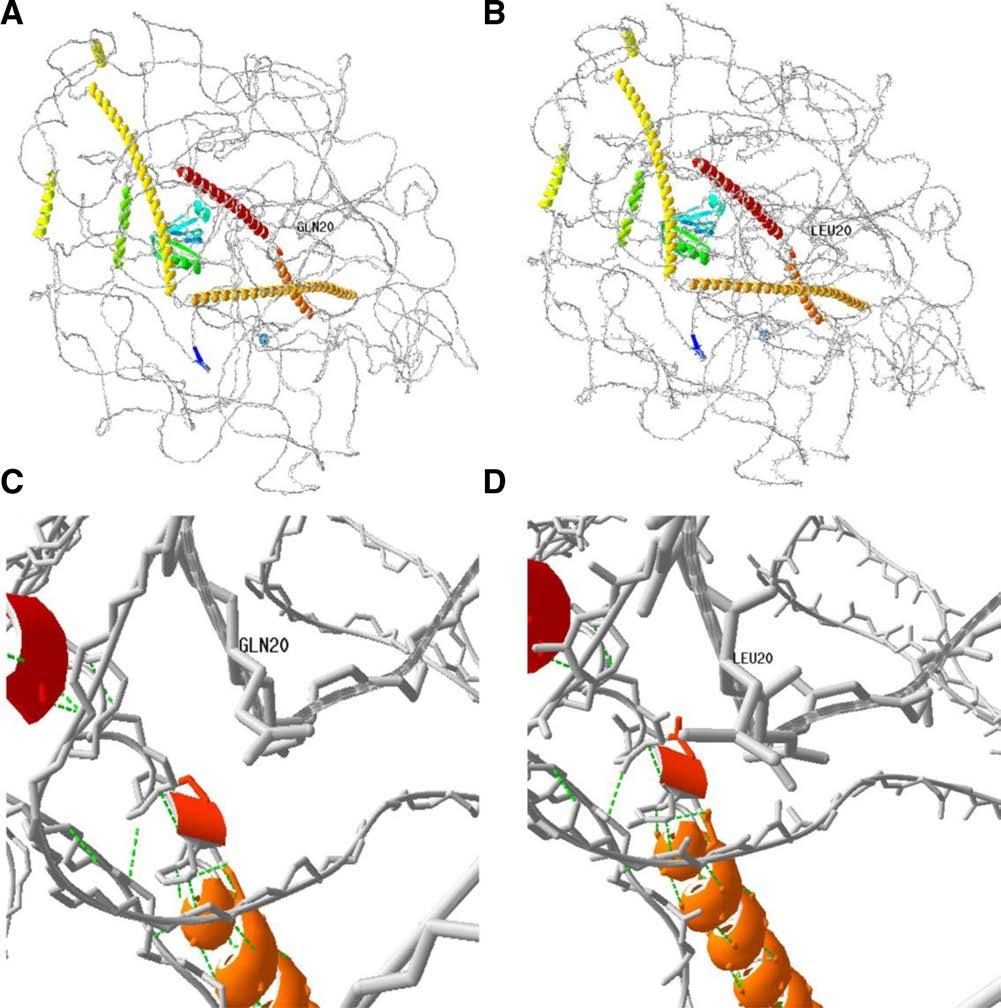
Difference between the wide-type and mutant protein (c.G59T) of TRIOBP (regional model). (A) Overall diagram of wild-type protein; (B) overall diagram of mutant protein; (C and D) details of wild protein. At position 20, the polar, hydrophilic, uncharged amino acid GLN is mutated to the nonpolar, hydrophobic amino acid LEU (as shown in D).

**Figure 4.**
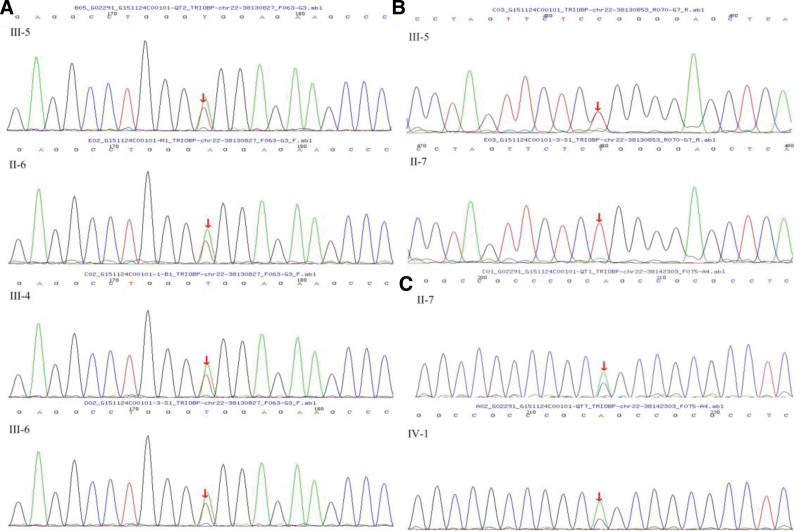
The results of pathogenic gene sequencing of the TRIOBP. (A) The c.A4484T mutation map, (B) The c.A4510G mutation map, and (C) The c.G59T mutation map.

Patient III-4 carries compound heterozygous mutations in the *TRIOBP* gene (NM_001039141): c.A4484T/p.E1495V and c.G59T/p.R20L. Patients II-6 and III-6 carry heterozygous mutations in the *TRIOBP* gene (NM_001039141): c.A4484T/p.E1495V. Protein structure homology modeling analysis revealed that the mutation at amino acid position 20 changed from a nonpolar hydrophobic GLN (Fig. 3A and C) to a nonpolar hydrophobic LEU (Fig. 3B and D). These changes in amino acid properties may affect the protein structure and consequently its function. Sanger sequencing confirmed that the c.A4484T mutation originated from the mother, while the c.A4510G and c.G59T mutations were not detected in other family members (Fig. 4).

In addition, patients II-6, III-4, and III-6 have compound heterozygous mutations in the *CHD7* gene: c.T2615C/p.I872T and c.3202-5T>C. Patients II-6 and III-4 also have compound heterozygous mutations in the *CHD23* gene: c.G5312A/p.R1771Q and c.C6250T/p.P2084. Sanger sequencing results of the family members were consistent with the results of whole-exome sequencing.

## 4. Discussion

Characteristics of autosomal recessive hereditary deafness *DFNB28* include prelingual sensorineural hearing loss. This condition is associated with mutations in the *TRIOBP* (Trio and F-actin-binding protein) gene, which has 3 transcripts known as TRIOBP-5, TRIOBP-4, and TRIOBP-1.^[8]^ Previous studies have shown that TRIOBP-4 and TRIOBP-5 proteins share the same upstream promoter in the first exon and are expressed in the upper and lower parts of the stereociliary rootlets of inner ear hair cells, respectively, participating in the formation of stereociliary rootlets.^[9,10]^ The basal part of stereociliary rootlets becomes thinner and extends into the electron-dense structures in the cuticular plate of hair cells, providing stability and rigidity against sustained mechanical pressure, thus playing an important role in maintaining the stability and rigidity of hair cell stereocilia.^[11]^ In this study, we identified novel pathogenic variants c.A4484T and c.A4510G in the *TRIOBP* gene in a family diagnosed with non-syndromic hearing loss. Sanger sequencing confirmed that these variants originated from the patient’s father and mother, respectively. The patient’s brother exhibited abnormal hearing phenotypes and carried a compound heterozygous mutation of the *TRIOBP* gene (c.A4484T/c.G59T), while the patient’s sister had an abnormal hearing phenotype and carried a heterozygous mutation of the *TRIOBP* gene (c.A4484T). The mother of the proband also carried a heterozygous mutation of the *TRIOBP* gene (c.A4484T), while the father carried a compound heterozygous mutation of the *TRIOBP* gene (c.A4510G/c.G59T) with the normal hearing phenotype. According to the audiological examination results, the hearing curve of the proband and his brother is similar, both of which are compound heterozygous mutation of *TRIOBP* gene. The same situation is also shown in patient II-6 and patient III-6, both carrying single heterozygous mutation of *TRIOBP* gene, and their hearing curve is similar. However, the relationship between these gene variations and the proband’s hearing loss has not been clinically confirmed. Pathogenic variations in the *TRIOBP* gene are usually associated with prelingual, moderate to severe hearing loss. However, there have also been reports of cases with postlingual hearing loss associated with pathogenic *TRIOBP* variations. In a Polish family with non-syndromic hearing loss, the proband was a 12-year-old patient with hearing loss, and genetic sequencing revealed compound heterozygous mutations in the *TRIOBP* gene as the cause of their deafness.^[12]^The study has found that the mutation is not a common cause of deafness, but for the post deaf people, the gene should also be thoroughly analyzed. Although pathogenicity analysis was performed according to the guidelines for variant interpretation issued by the American Society of Medical Genetics and Genomics (ACMG): c.A4484T, c.A4510G were suspected benign variants using evidence of BP4-Moderate.We still believe that the c.A4484T/c.A4510G mutation in the TRIOBP gene may be a pathogenic variant of hearing loss in the proband. It is known that the R1 motif of TRIOBP-5/-4 is the major actin-binding domain. There are also irregular actin-binding sites in the CC domains or PH domains in TRIOBP-5/-1. In the proband’s mutation sites, the amino acid at position 1495 changed from a polar hydrophilic glutamate (GLU) to a nonpolar hydrophobic valine (VAL), and the amino acid at position 1504 changed from a polar positively charged arginine (ARG) to a polar uncharged glycine (GLY). These changes in amino acid properties may affect protein structure and, therefore, its function, leading to hearing loss. However, the existing experimental data are not enough to prove this view, and further analysis of protein function should be needed in the later stage to verify this view.

Through whole-exome sequencing, compound heterozygous mutations (c.T2615C and c.3202-5T>C) in the *CHD7* gene and compound heterozygous mutations (c.G5312A and c.C6250T) in the *CDH23* gene were found in the proband’s brother. Sanger sequencing confirmed that the mother carried compound heterozygous mutations (c.T2615C and c.3202-5T>C) in the *CHD7* gene and compound heterozygous mutations (c.G5312A and c.C6250T) in the *CDH23* gene, while the sister carried compound heterozygous mutations (c.T2615C and c.3202-5T>C) in the *CHD7* gene. The father did not carry the aforementioned gene mutations, it is shown that the 2 mutation sites of CHD 7 and the 2 mutation sites of CDH 23 are located on the same chromosome of chromosome 8 and chromosome 10, respectively (Table 2). *CHD7* gene mutations are the main cause of CHARGE syndrome,^[13]^ and the *CDH23* gene, which encodes otocadherin, plays an important role in the structure and function of hair cell stereocilia.^[14]^ Mutations in the *CDH23* gene can lead to non-syndromic hearing loss (NSHL) with only auditory manifestations or to Usher syndrome type 1D (USH1D) characterized by hearing loss, retinal pigment deposits, and vestibular dysfunction. *CDH23* gene mutations generally follow an autosomal recessive inheritance pattern, where carriers of a single heterozygous mutation do not develop the disease, but individuals carrying 2 or more compound heterozygous mutations may manifest as NSHL or Usher syndrome.^[15]^ Mutations in the *CHD7* and *CDH23* genes are commonly associated with hearing loss and typically occur in an autosomal recessive manner. However, there are still differences in the types of mutations in the *CHD7* gene and the clinical presentations of *CDH23* gene mutations. Common mutation types in the *CHD7* gene include nonsense mutations, frameshift mutations, splice site mutations, and missense mutations, among others. The results of domestic studies slightly differ from those of international studies. In the Chinese population with CHARGE syndrome, frameshift mutations in the *CHD7* gene, including c.6292C>T, c.7957C>T, c.718C>T, c.5883C>T, and c.2966G>A, have been reported. In this study, we identified compound heterozygous mutations (c.T2615C and c.3202-5T>C) in the *CHD7* gene and compound heterozygous mutations (c.G5312A and c.C6250T) in the *CDH23* gene, which have not been reported as clinically pathogenic. Therefore, the possibility of *CHD7* and *CDH23* gene mutations causing the clinical phenotypes in patients II-6, III-4, and III-6 is relatively low.

**Table 2:** Results of nuclear gene testing.

Case number	Mutated gene	Mutation position	Nucleotide change	Source of variation
II-6	CHD7	chr8-61732567 chr8-61736394	c.T2615C c.3202-5T>C	–
	CDH23	chr10-73539148 chr10-73551089	c.G5312A c.C6250T	–
	TRIOBP	chr22-38130827	c.A4484T	–
II-7	TRIOBP	chr22-38130853 chr22-38142303	c.A4510G c.G59T	–
III-4	CHD7	chr8-61732567 chr8-61736394	c.T2615C c.3202-5T>C	Mother Mother
	CDH23	chr10-73539148 chr10-73551089	c.G5312A c.C6250T	Mother Mother
	TRIOBP	chr22-38130827 chr22-38142303	c.A4484T c.G59T	Mother Father
III-5	TRIOBP	chr22-38130827 chr22-38130853	c.A4484T c.A4510G	Mother Father
III-6	CHD7	chr8-61732567 chr8-61736394	c.T2615C c.3202-5T>C	Mother Mother
	TRIOBP	chr22-38130827	c.A4484T	Mother

In summary, this study first found a new suspected deafness pathogenic locus (c.A4484T and c.A4510G) in the *TRIOBP* gene in a family diagnosed with non-syndromic hearing loss, which provided new clues for the genetic variation of this family. Future research can further explore the pathogenic mechanisms of these gene mutations and investigate the functional aspects of the corresponding genes. Further studies are needed to determine the etiology of hearing loss in patients II-4, III-4, and III-6.

## Acknowledgments

We thank the many individuals with Non-Syndromic Deafness and their families who participated in the research studies cited in this report.

## Author contributions

**Data curation:** Hong Zhou, Gang Guo.

**Formal analysis:** Hong Zhou, Gang Guo, Jianjun Gao, Hong Duan.

**Funding acquisition:** Hong Duan.

**Investigation:** Jianjun Gao.

**Software:** Jianjun Gao.

**Supervision:** Gang Guo.

**Validation:** Hong Zhou.

**Writing – original draft:** Hong Zhou, Gang Guo.

**Writing – review & editing:** Gang Guo, Hong Duan.
